# Supplementary figures and images for: Phylogenetic relationships of the genus Mischonyx Bertkau, 1880, with taxonomic changes and three new species description (Opiliones: Gonyleptidae)
Source: PeerJ. 2021 Sep 28;9:e11682. doi: 10.7717/peerj.11682 (PMC8485841; doi:10.7717/peerj.11682)

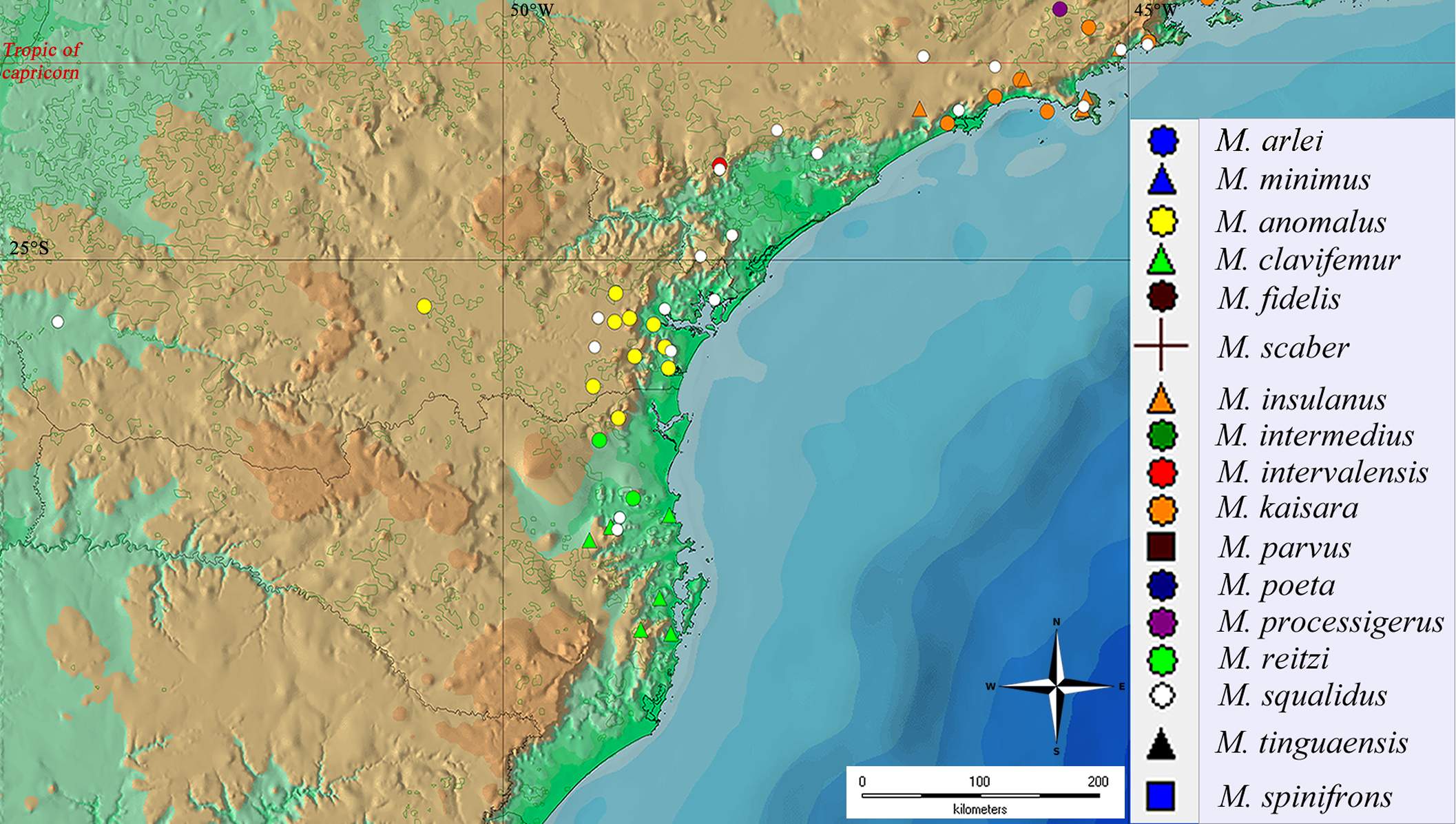

Supplement: Supplemental Information 9 — Legends are in the right of the figure. The black grid represents the full meridians and parallels. [file peerj-09-11682-s009.png]

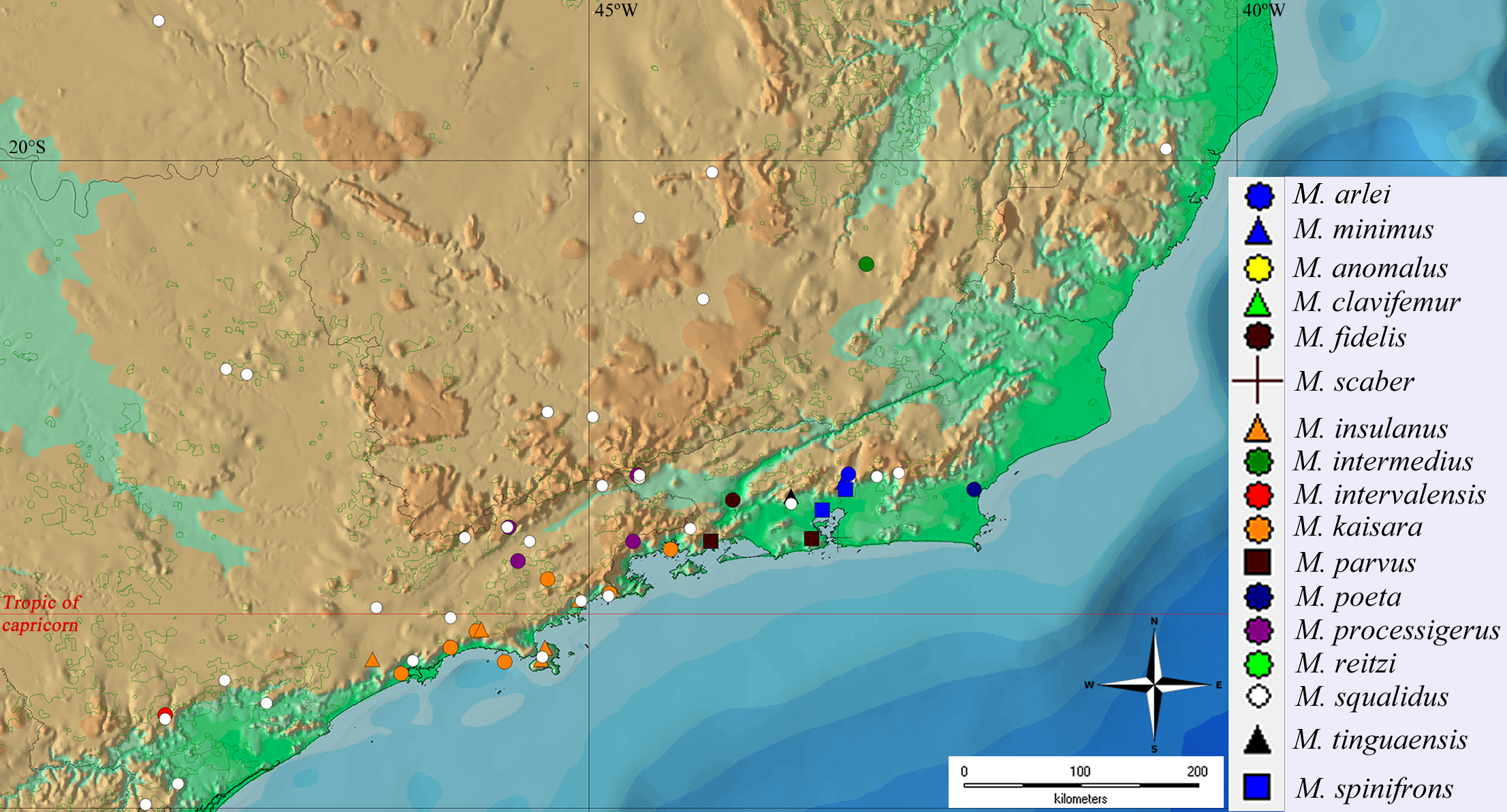

Supplement: Supplemental Information 10 — Legends are in the right of the figure. The red line represents the Tropic of Capricorn and the black grid represents the full meridians and parallels. [file peerj-09-11682-s010.png]

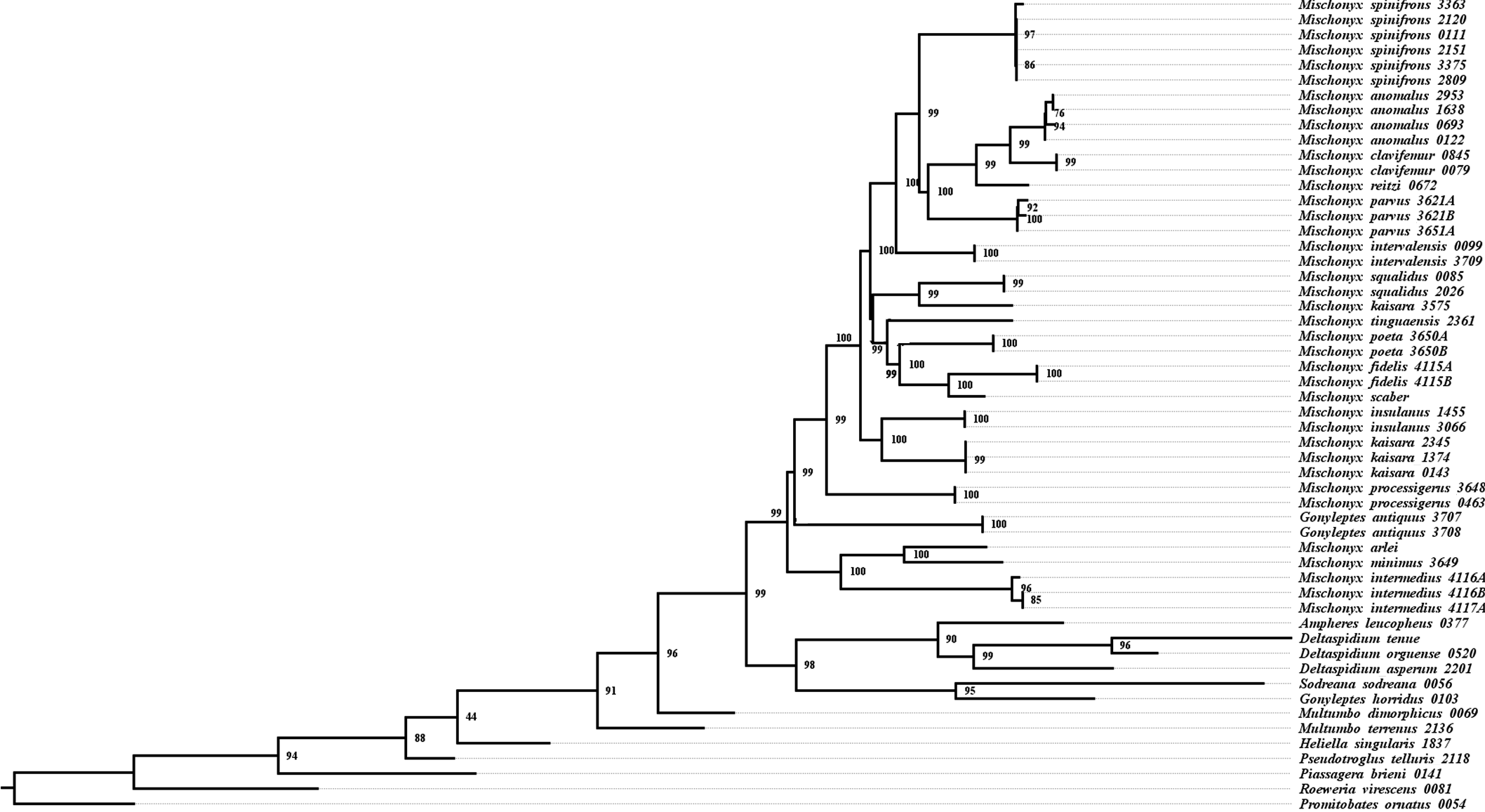

Supplement: Supplemental Information 11 — The values near the nodes are the Bootstrap values of each one. Numbers after the species name are the LAL Vouchers of each individual. [file peerj-09-11682-s011.png]

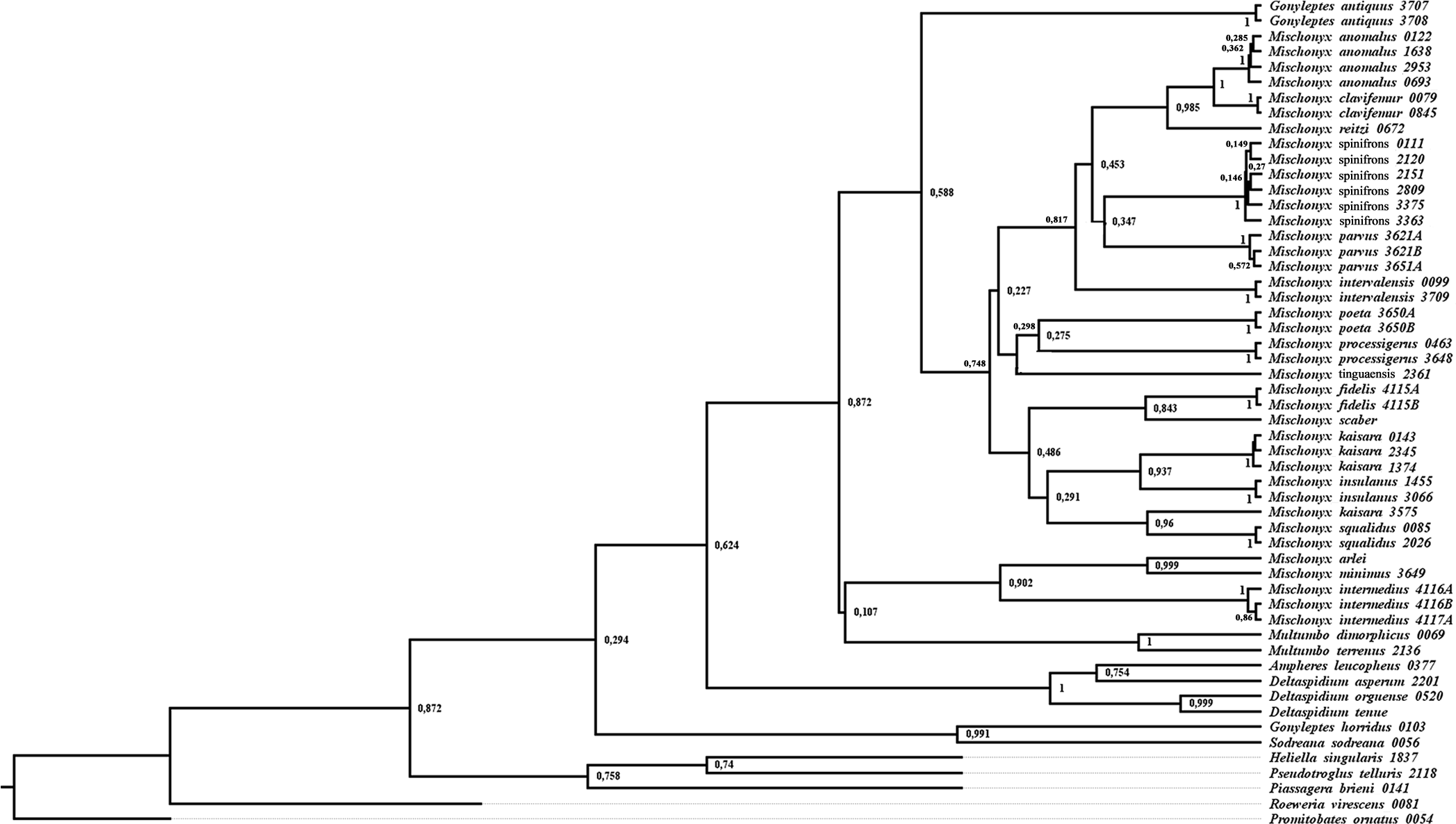

Supplement: Supplemental Information 12 — The values near the nodes are the posterior probability of each one. Numbers after the species name are the LAL Vouchers of each individual. [file peerj-09-11682-s012.png]

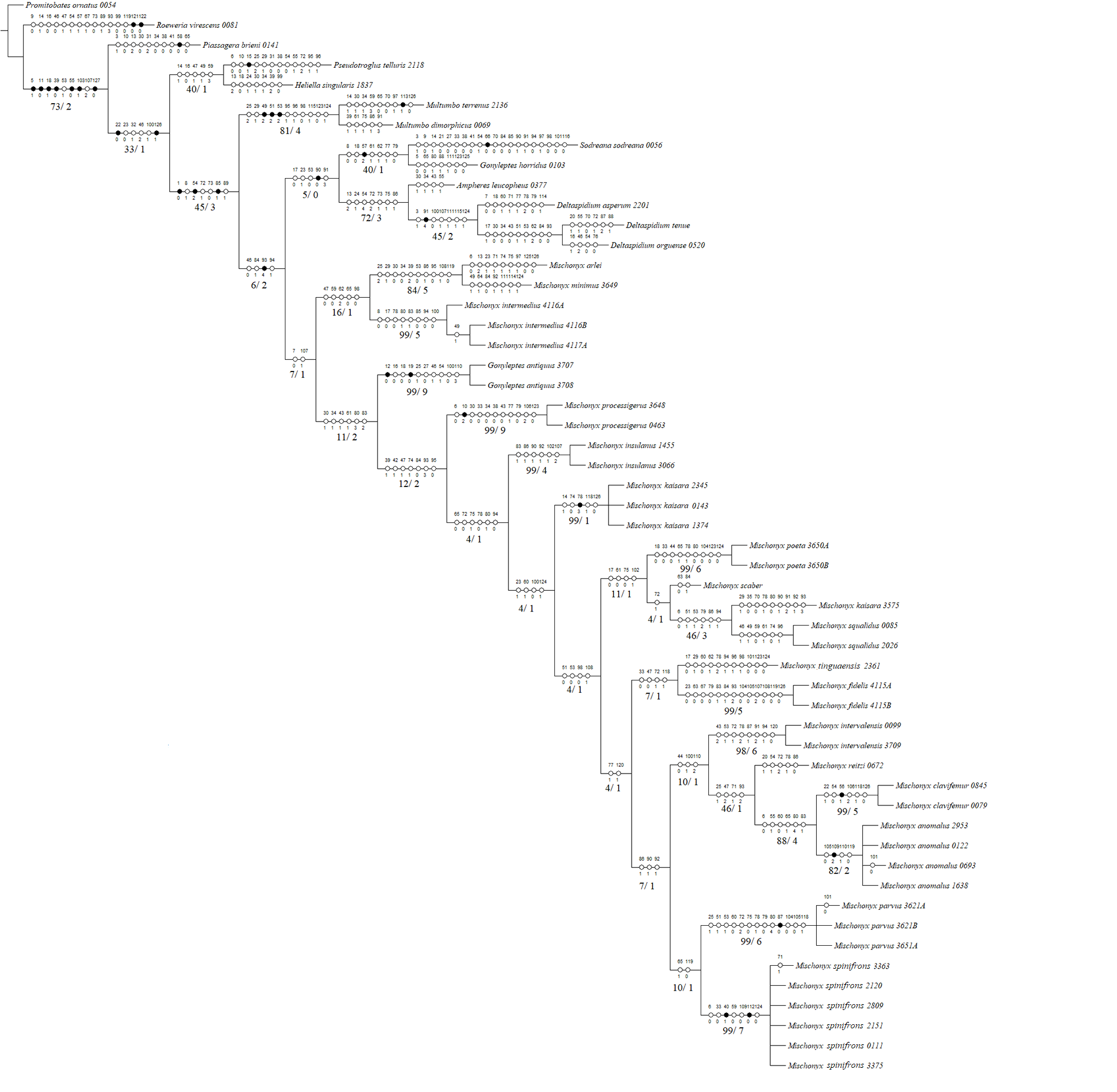

Supplement: Supplemental Information 13 — The values near the nodes are the Bootstrap/ Bremer values of each one. The circles in each node represent the unambiguous changes only. Black circles represent non homoplastic and empty circles represent homoplastic synapomorphies. Numbers after the species name are the LAL Vouchers of each individual. [file peerj-09-11682-s013.png]

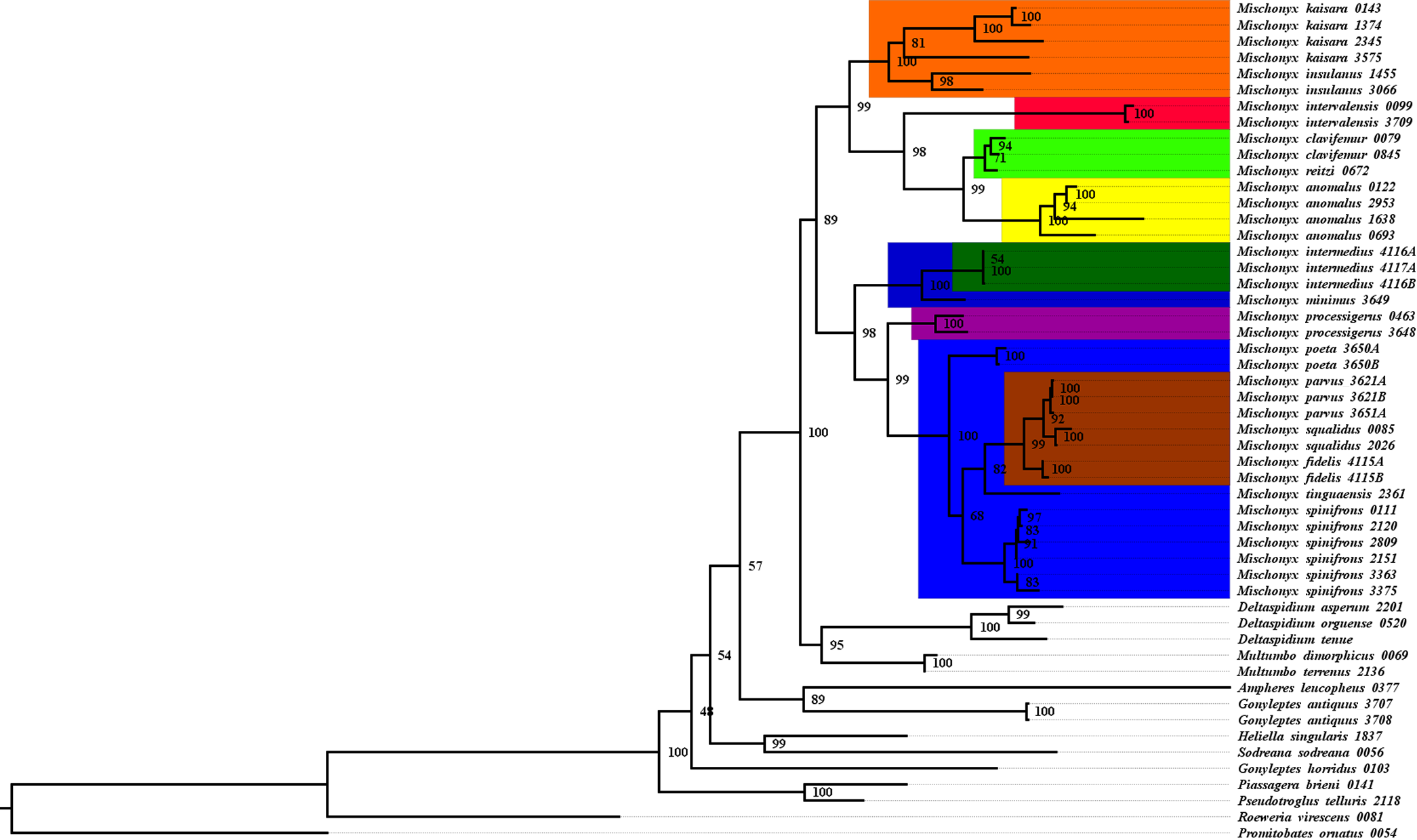

Supplement: Supplemental Information 14 — The values near the nodes are the bootstrap values of each one. Numbers after the species name are the LAL Vouchers of each individual. The colored clades are according to their location, respective to each Area of Endemism. Light green: SC; yellow: PR; Red: SSP; orange: SMSP; blue: Org; dark green: Esp; purple: Boc; brown: LSRJ and M. squalidus. [file peerj-09-11682-s014.png]

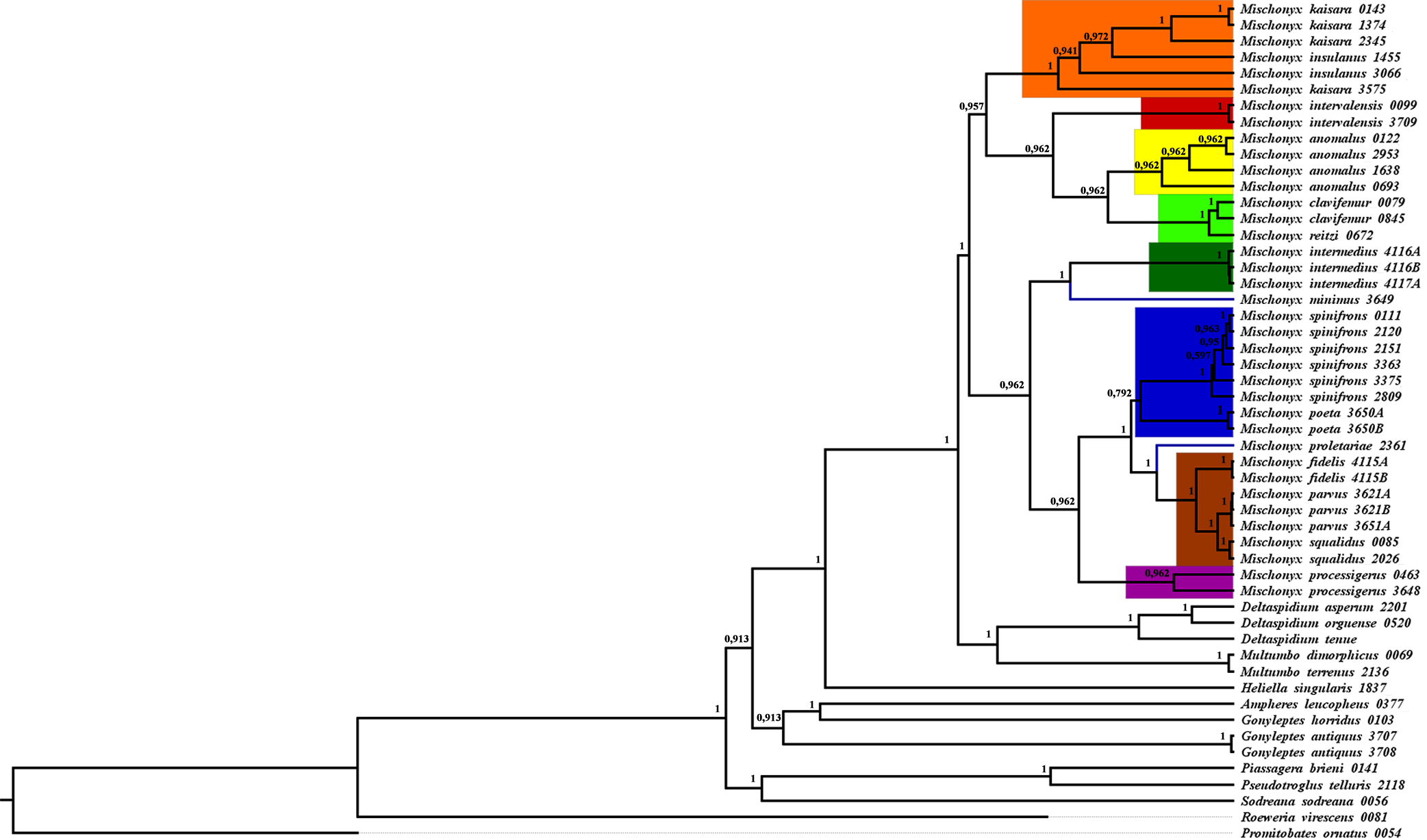

Supplement: Supplemental Information 15 — The values near the nodes are the posterior probability of each one. Numbers after the species name are the LAL Vouchers of each individual. The colored clades are according to their location, respective to each Area of Endemism. Light green: SC; yellow: PR; Red: SSP; orange: SMSP; blue: Org; dark green: Esp; purple: Boc; brown: LSRJ and M. squalidus. [file peerj-09-11682-s015.png]

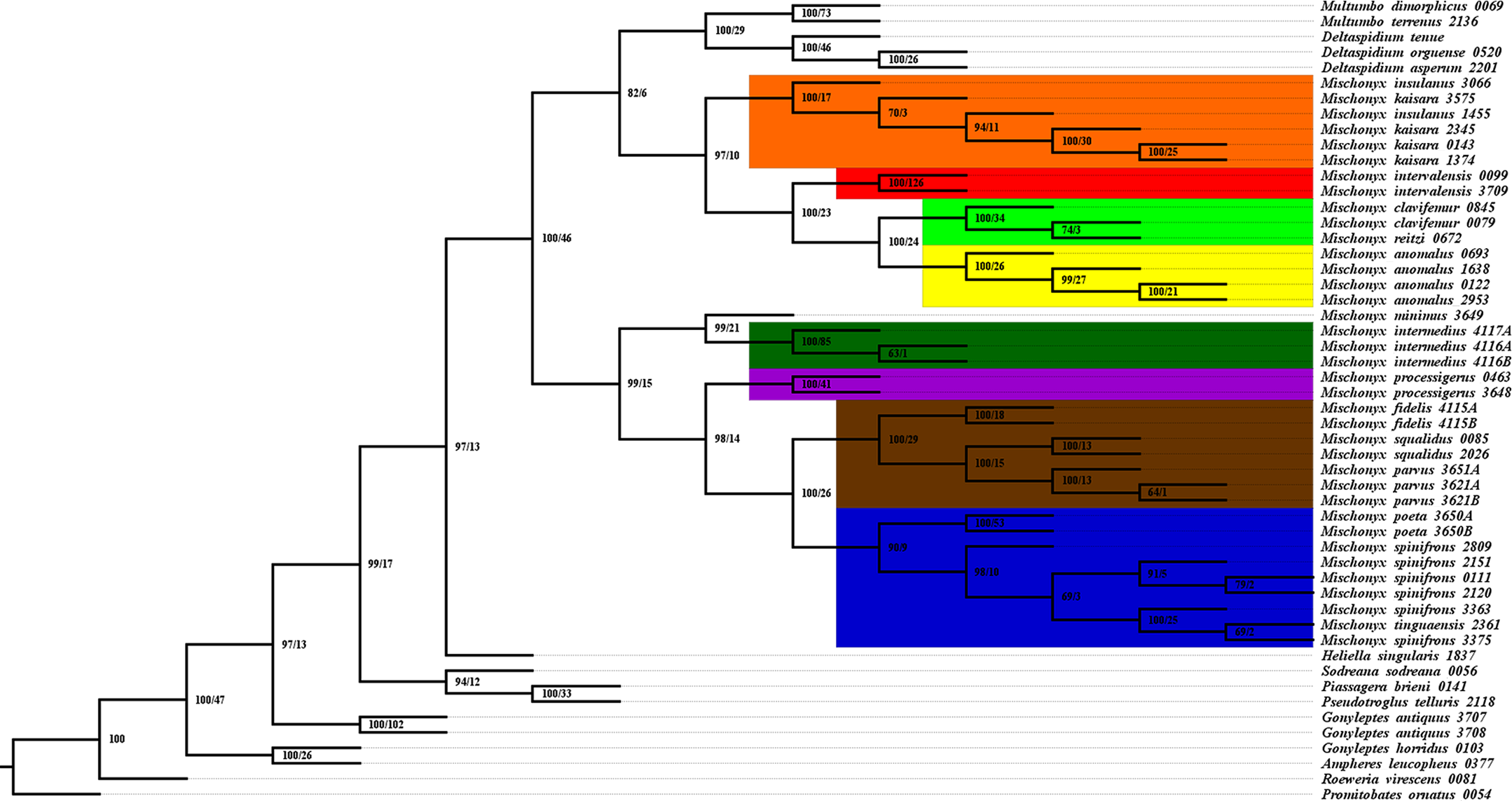

Supplement: Supplemental Information 16 — The values near the nodes are the bootstrap values of each one. Numbers after the species name are the LAL Vouchers of each individual. The colored clades are according to their location, respective to each Area of Endemism. Light green: SC; yellow: PR; Red: SSP; orange: SMSP; blue: Org; dark green: Esp; purple: Boc; brown: LSRJ and M. squalidus. [file peerj-09-11682-s016.png]

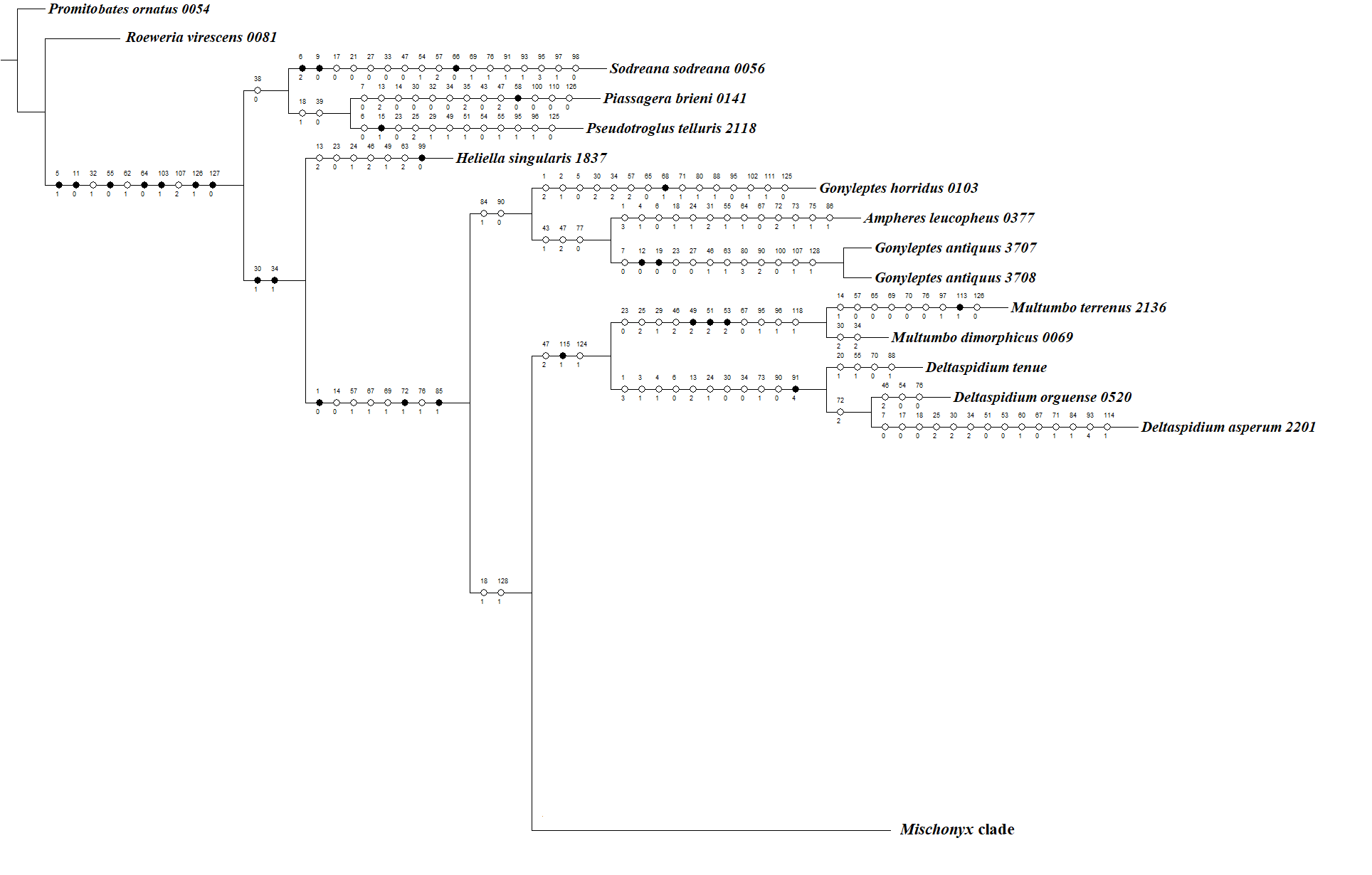

Supplement: Supplemental Information 17 — The circles in each node represent the unambiguous changes only. Black circles represent non homoplastic and empty circles represent homoplastic synapomorphies. Numbers after the species name are the LAL Vouchers of each individual. [file peerj-09-11682-s017.png]

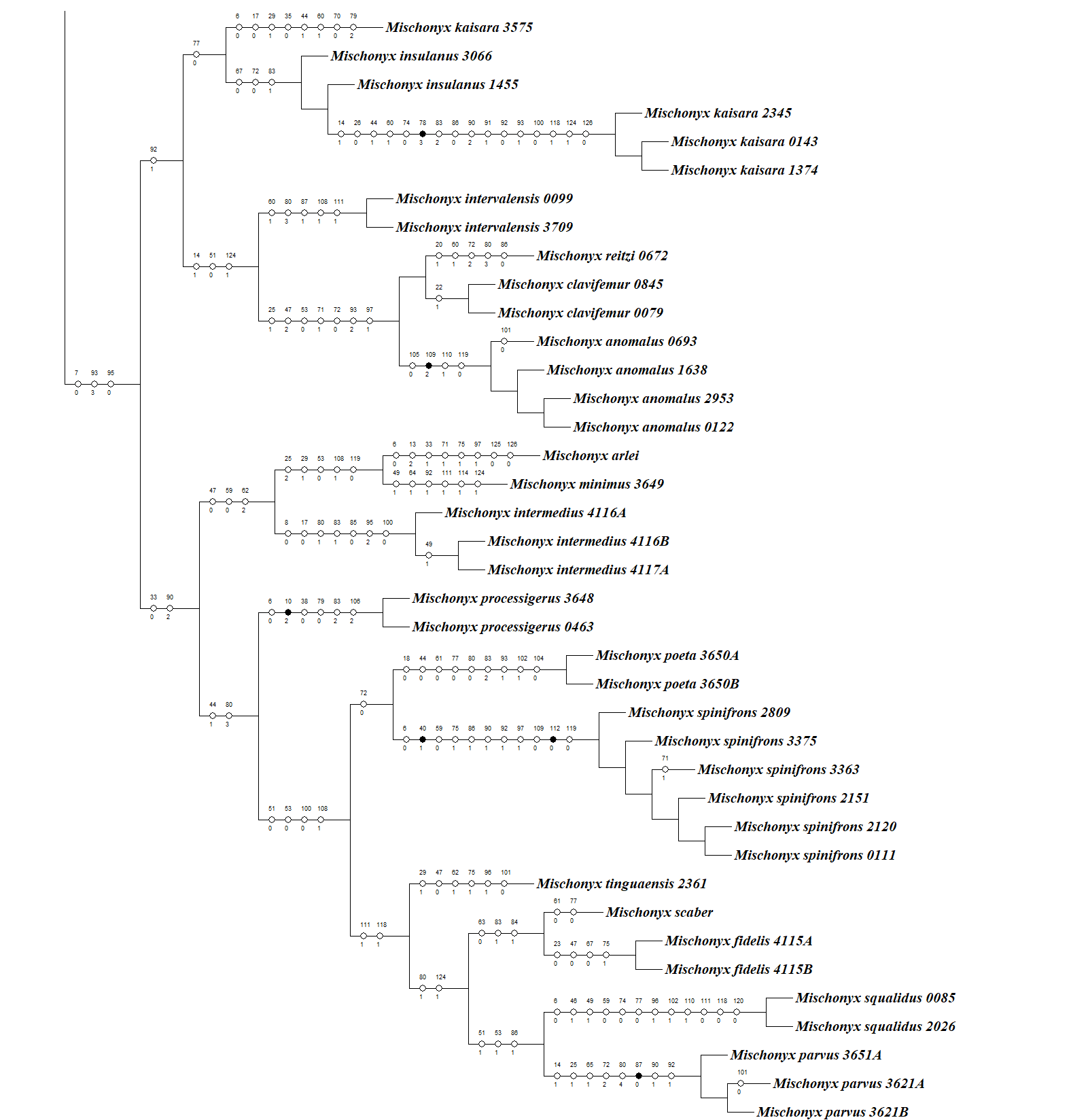

Supplement: Supplemental Information 18 — The circles in each node represent the unambiguous changes only. Black circles represent non homoplastic and empty circles represent homoplastic synapomorphies. Numbers after the species name are the LAL Vouchers of each individual. [file peerj-09-11682-s018.png]

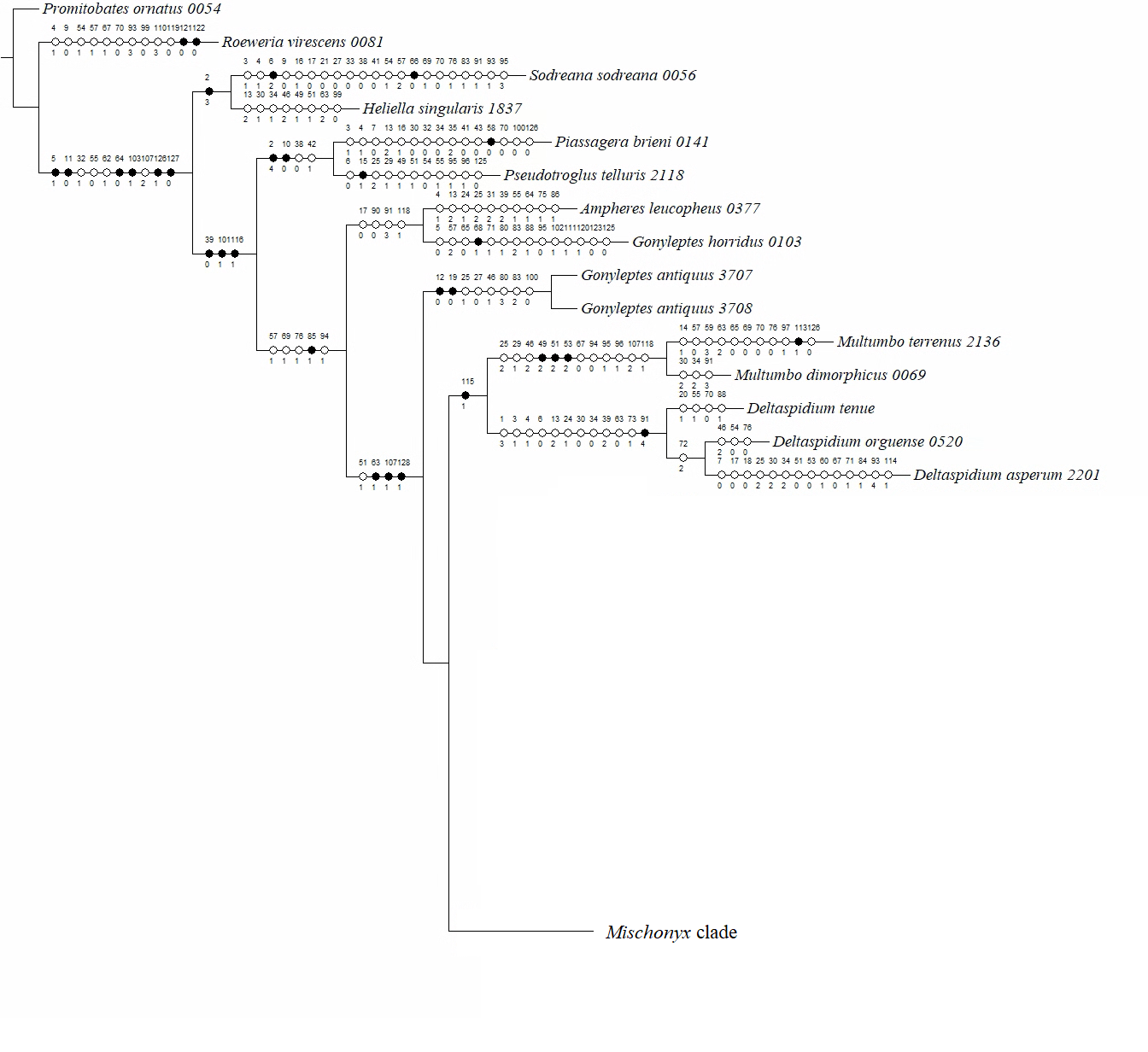

Supplement: Supplemental Information 19 — The circles in each node represent the unambiguous changes only. Black circles represent non homoplastic and empty circles represent homoplastic synapomorphies. Numbers after the species name are the LAL Vouchers of each individual. [file peerj-09-11682-s019.png]

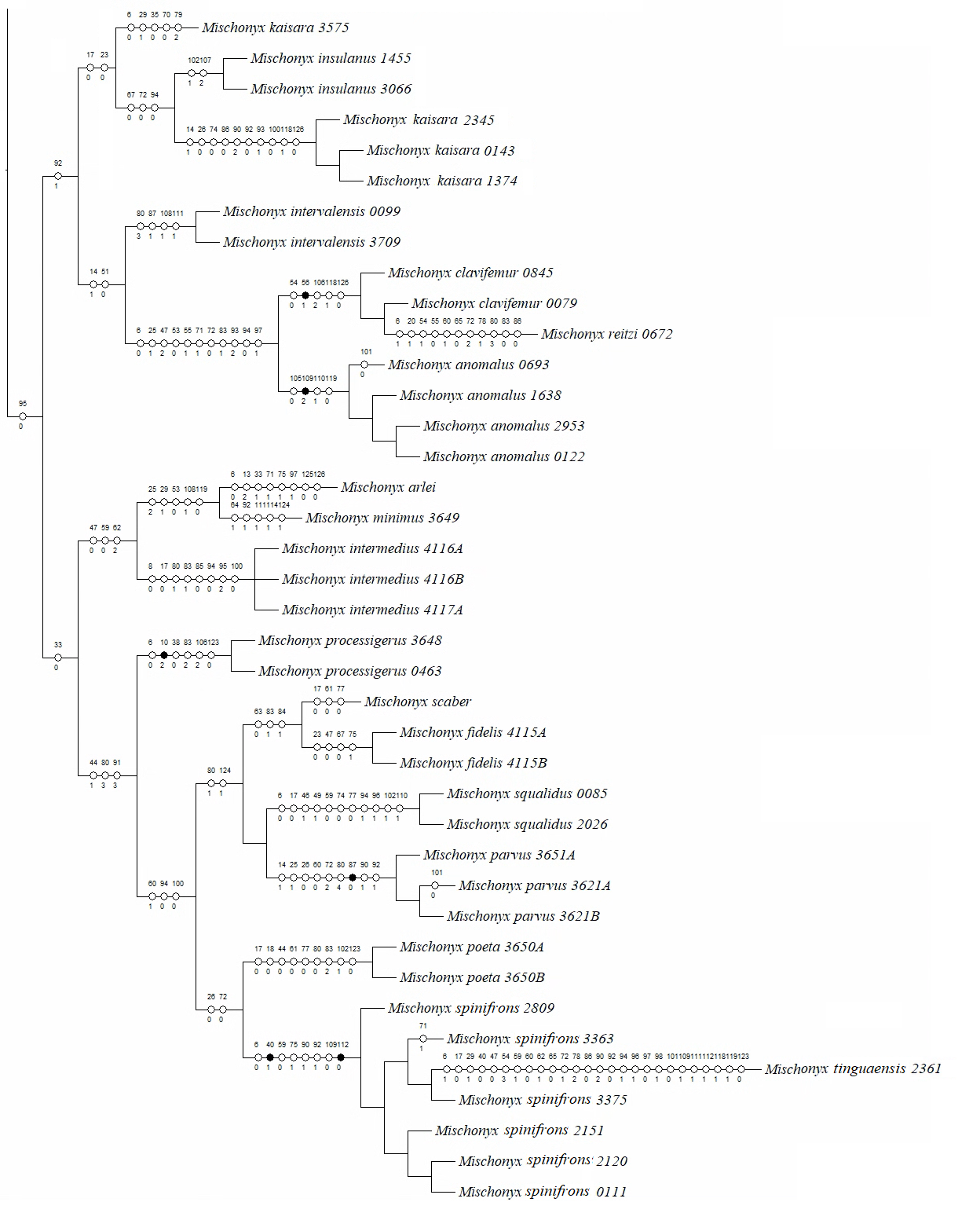

Supplement: Supplemental Information 20 — The circles in each node represent the unambiguous changes only. Black circles represent non homoplastic and empty circles represent homoplastic synapomorphies. Numbers after the species name are the LAL Vouchers of each individual. [file peerj-09-11682-s020.png]
